# Effect of breastfeeding for 6 months on disease outcomes in patients with Kawasaki disease

**DOI:** 10.1371/journal.pone.0261156

**Published:** 2021-12-21

**Authors:** Mindy Ming-Huey Guo, I-Hsin Tsai, Ho-Chang Kuo

**Affiliations:** 1 Department of Pediatrics, Kaohsiung Chang Gung Memorial Hospital and Chang, Gung University College of Medicine, Kaohsiung, Taiwan; 2 Kawasaki Disease Center, Kaohsiung Chang Gung Memorial Hospital and Chang, Gung University College of Medicine, Kaohsiung, Taiwan; 3 Department of Family Medicine, Kaohsiung Chang Gung Memorial Hospital and Chang, Gung University College of Medicine, Kaohsiung, Taiwan; Kaohsuing Medical University Hospital, TAIWAN

## Abstract

**Background:**

Kawasaki disease (KD) is a systematic vasculitis that occurs predominantly in young children, and is the leading cause of acquired heart disease in children younger than five-years-old in developed countries. Although the etiology of KD is unknown, it is believed to be an inflammatory disease resulting from abnormal immune responses to possible environmental or infectious stimuli in genetically predisposed individuals. Breast milk contains numerous anti-inflammatory factors which may protect against allergic and autoimmune diseases. In this study we tried to examine the effect of breastfeeding for 6 months or more on disease outcomes in patients with Kawasaki disease.

**Methods:**

A retrospective cohort study of 249 KD patients admitted from 1999- 2013 who were older than 6 months at time of diagnosis and had data regarding breastfeeding in the first 6 months of life. Demographic, clinical and laboratory data was collected by chart review. Continuous data was compared using Student’s t-test and categorical variables were compared using Chi-square. Stepwise multivariate regression of all demographic factors was performed.

**Results:**

Breastfeeding for 6 months or more was associated with a shorter total duration of fever (5.980± 1.405 Vs. 6.910 ± 2.573 days, *p* = 0.001) and a lower risk of developing persistent coronary artery lesions (CALs) (7.8% Vs. 20.2%, *p*-value = 0.039) on univariate analysis. Multivariate regression of all factors associated with CALs including breastfeeding for 6 months found that only the presence of CALs at baseline (β-coefficient = 0.065, p < 0.001) and white blood count (β-coefficient = 0.065, p = 0.018) remained significant after regression analysis.

**Conclusions:**

Breastfeeding for 6 months or more was associated with a shorter duration of fever and a lower risk of persistent CAL formation in patients with KD on univariate analysis, although this effect may be modest when other factors such as the presence of CALs at baseline and white blood cell count are also taken into consideration.

## Introduction

Kawasaki disease (KD) is an acute, potentially fatal vasculitis that occurs predominantly in young children [1], and is the leading cause of acquired heart disease in children younger than five-years-old in developed countries. Despite advances in immunomodulation and antithrombotic treatment, coronary arterial complications still occur in 10%-15% of patients [1–5]. The incidence of KD has been reported to be higher in Asian countries when compared with Western populations. In Taiwan, the incidence of KD is the third-highest worldwide and ranges from 50 to 82.8 per 100,000 children; the only countries with a higher incidence are Japan (264.8/100,000) and Korea (127.7/100,000) [6].

Breastfeeding, a natural and nutritious solution for feeding the infant, has many health benefits, including protection against gastrointestinal and respiratory infections. Breast milk also contains numerous factors that modulate and promote development of the immune system during infancy [7], including polyunsaturated long-chain fatty acids and interleukin-10 [8], which promote anti-inflammatory responses and may protect against allergic and autoimmune diseases. For example, animal studies have found that prolonged breastfeeding in rats is associated with higher levels of anti-inflammatory regulatory T cells and a decreased risk of developing autoimmune diabetes [9]. Newborns who receive breast milk with lower levels of polyunsaturated long-chain fatty acids have been found to have a higher risk of atopic disease by 18 months of age [10]. Epidemiologic studies have also found that increased duration of breastfeeding is associated with lower risks of atopic disease such as asthma, eczema and allergic rhinitis [11] and autoimmune disease such as juvenile idiopathic arthritis [12].

Although the etiology of KD is unknown, it is believed to be an inflammatory disease resulting from abnormal immune responses to possible environmental or infectious stimuli in genetically predisposed individuals [13]. Identification of possible triggering agents remain elusive. Retrospective studies have found up to 30% of KD patients also have concurrent infections, although a broad-spectrum of both viral and bacterial agents have been identified [14]. Similarly, in a systemic review of KD and immunization revealed several vaccines appeared to have a temporal relationship to the onset of KD, although overall the incidence rate was quite low. Vaccinations with a possible temporal relationship to KD include but are not limited to pneumococcal vaccines, rotavirus vaccine, meningococcal vaccine etc. [15].

Because breastfeeding promotes anti-inflammatory responses and has been shown to protect against both allergic and autoimmune disease, it is plausible that breastfeeding may also protect against KD. Indeed, a recent large-scale population based study of Japanese children has shown that breastfeeding confers protection against the development of KD [16].

Due to the multiple health benefits of breastfeeding, the American Academy of Pediatrics currently recommends breastfeeding for at least six months after birth [17]. In light of these guidelines, we performed a retrospective cohort study to examine whether breastfeeding for 6 months or more after birth had any impact on major disease outcomes in patients with KD, including intravenous immunoglobulin (IVIG) resistance, total duration of fever, and the development of coronary artery lesions (CALs).

## Material and methods

Our study design was approved by the Institutional Review Board of Kaohsiung Chang Gung Memorial Hospital. Data was obtained from medical records of patients admitted to Kaohsiung Chang Gung Memorial hospital. Patients were admitted from 1997 to 2013. Data was accessed from 2017 to 2018. Data was not fully anonymous when accessed.

In this retrospective cohort study, we reviewed all children enrolled in our Kawasaki Disease Registry from 1999 to 2013 at Kaohsiung Chang Gung Memorial Hospital in Taiwan. Because the aim of our study was to examine the effect of breastfeeding for 6 months or more on the clinical outcomes on KD, we included only those who developed KD after 6 months of age, and had data regarding breastfeeding for the first 6 months of life on chart review. In total 249 patients were included in our analysis. All patients in this study fulfilled the American Heart Association diagnostic criteria for KD, namely fever for over five days and at least four out five clinical symptoms including oral mucosal changes, bilateral non-suppurative conjunctivitis, cervical lymphadenopathy of > 1.5 cm in diameter, changes in the peripheral extremities and polymorphous rash [18]. All patients received at least one dose of 2g/kg of IVIG after diagnosis of KD, and those with IVIG resistance (defined as persistent fever 48 hours after initial IVIG therapy) were given a second dose of IVIG three days after the first.

Medical records were reviewed for age, gender, total duration of fever, duration of breastfeeding, maternal age at patient birth, day of fever the first dose IVIG therapy was given, signs of mucocutaneous inflammation and presence of IVIG resistance. Total duration of fever was calculated by counting the number of days beginning from the first day of fever to the last day that fever was recorded, and included days of recurrent fever in patients who were IVIG resistant. Laboratory data that was collected 24 hours prior to IVIG therapy was also analyzed, including baseline white blood cell count with differential (segmented neutrophil, lymphocyte, monocyte and eosinophil percentages), platelet count, presence of pyuria on urine analysis, hemoglobin, alanine aminotransferase, aspartate aminotransferase, C-reactive protein and albumin levels. Echocardiography was performed using a Philips iE33 ultrasound machine by a pediatric cardiologist with at least two years of ultrasound experience. Luminal diameters were measured at the following segments: the left main coronary artery (LMCA) was measured between the ostium and the first bifurcation; the left anterior descending artery (LAD) was measured distal to and away from the bifurcation of the branch from the LCA, and the right coronary artery (RCA) luminal diameter was measured at the straight section of the artery immediately after the rightward turn of the artery as previously described [19]. Transthoracic echocardiogram was performed at the time of diagnosis, and then followed at least four time points: one week, one month, two months and six months after diagnosis.

The internal diameter of the coronary arteries were measured and patients were defined as having coronary arteries lesions (CALs) if any of the following findings were found to persist at least one month after disease onset: if the internal lumen diameter of the coronary arteries were ≥3 mm in children younger than 5 years old or ≥4 mm in children older than 5 years old, or if the coronary artery lumen was clearly irregular [2,20]. KD patients with coronary artery ectasia or dilation that disappeared within the initial 4 weeks after the onset of illness were defined as transient ectasia and were not judged as persistent CALs. We also reviewed chart and echocardiography data for other manifestations of cardiac inflammation, including myocarditis, pericarditis, pericardial effusion or shock.

Patients were split into two groups, those with were breastfed for 6 months or more, and those who received less than 6 months of breastfeeding. Chi-squared test was used to compare categorical data. The Shapiro-Wilk test was used to check the normality of continuous data; data which conformed to a normal distribution were compared using the Student’s *t-*test, while data which was found to be skewed were compared using the Mann-Whitney U test. In addition, to identify possible factors associated with coronary artery involvement within our data set, we also divided patients into to those with coronary involvement and those without, and performed a univariate analysis of all demographic, echocardiographic and laboratory data to identify individual factors associated with the development of coronary artery lesions persisting for more than one month. Factors which were statistically significant (ie. which had a *p*-value of < 0.005) were than included in our multivariate logistic regression model. A p-value <0.05 was considered statistically significant. The statistical software package SPSS for Windows, version 14.0 (SPSS Inc., Chicago,IL, USA) was used for all statistical analysis.

## Results

In total 249 KD patients were analyzed, of which 23 patients (9.2%) had IVIG resistance, 31 patients (12.5%) had CALs at baseline prior to IVIG therapy (11 of which resolved within one month of disease onset) and 44 patients (17.7%) had CALs which persisted for at least one month or more after disease onset. Fifty-one patients were breastfed for 6 months or more after birth (20.5%), while the remaining 198 patients were breastfed for less than 6 months after birth (79.5%). There was no difference in demographic variables including age, sex, maternal age at patient birth, or day of fever IVIG was given between those who received 6 months or more of breastfeeding after birth and those who did not (Table 1).

**Table 1:** Comparison of demographic and clinical data between KD patients with more than 6 months of breastfeeding and those without.

	Breastfeeding for < 6 months (N = 198)	Breastfeeding for ≥ 6 months (N = 51)	*p*-value
Male Sex	120/198 (60.6%)	33/51 (64.7%)	0.592
Age at time of KD diagnosis (years)	1.991 ± 1.731 (N = 198)	1.908 ± 1.618 (N = 51)	0.757
Maternal age at patient birth (years)	29.97 ± 4.558 (N = 192)	31.40 ± 4.341 (N = 48)	0.052
Day of fever IVIG was given (days)	6.02 ± 2.255 (N = 193)	5.66 ± 1.349 (N = 50)	0.152
Total duration of fever (days)	6.910 ± 2.573 (N = 195)	5.980 ± 1.405 (N = 50)	0.001*
Non-suppurative conjunctivitis	122/198 (61.6%)	41/51 (80.4%)	0.012*
Oral mucosa changes	172/198 (86.9%)	47/51 (92.2%)	0.301
Lymphadenopathy	35/198 (17.7%)	14/51 (27.5%)	0.117
Polymorphous rash	163/198 (82.3%)	44/51 (86.3%)	0.502
Changes in the hands and feet	132/198 (66.7%)	35/51 (68.6%)	0.790
Erythema around BCG site	41/168 (24.4%)	20/41 (48.8%)	0.002*
IVIG resistance	20/198 (10.1%)	3/51 (5.9%)	0.430
Pericardial effusion	5/198 (2.5%)	3/51 (5.9%)	0.211
Coronary artery lesion at baseline	28/198 (14.1%)	3/51 (5.9%)	0.111
Coronary artery lesion persisting for ≥ 1 month	40/198 (20.2%)	4/51 (7.8%)	0.039*

All continuous lab values are presented as mean ± standard deviation. An asterisk denotes *p*-values of < 0.05. BGC: Bacillus Calmette-Guérin, IVIG: Intravenous immunoglobulin.

KD patients who received more than 6 months of breastfeeding were more likely to present with non-suppurative conjunctivitis (80.4% Vs. 61.6%, *p* = 0.012) and Bacillus Calmette-Guérin (BCG) inoculation site erythema (48.8% Vs. 24.4%, *p* = 0.002). Breastfeeding for more than 6 months after birth was associated with a shorter total duration of fever (5.980 ± 1.450 Vs. 6.910 ± 2.573 days, mean ± SD, *p*= 0.001), and a lower risk of developing CALs persisting for one month or more (7.8% Vs. 20.2%, *p*-value = 0.039). CAL formation at baseline prior to IVIG therapy and IVIG resistance was not associated with differences in breastfeeding (Table 1). Breastfeeding for more than 6 months was also associated with higher albumin levels prior to IVIG therapy (3.571 ± 0.546 Vs. 3.256 ± 0.648 g/dL, mean ± SD, *p* = 0.006, Table 2), but was not associated with any other differences of initial laboratory data prior to IVIG therapy. Only eight patients were found to have pericardial effusion on echocardiogram at diagnosis, but none of our patients had signs of myocarditis, pericarditis or shock.

**Table 2:** Comparison of laboratory data between KD patients with more than 6 months of breastfeeding and those without 24 hours prior to IVIG therapy.

	Breastfeeding for < 6 months (N = 198)	Breastfeeding for ≥ 6 months (N = 51)	*p*-value
White cell count (1000/cmm^3^)	13.809 ± 4.756 (N = 194)	13.892 ± 4.986 (N = 49)	0.817
Hemoglobulin (g/dL)	11.049 ± 1.136 (N = 194)	10.871 ± 1.333 (N = 49)	0.347
Platelet count (1000/cmm^3^)	323.129 ± 131.220 (N = 194)	340.837 ± 110.861 (N = 49)	0.507
Segmented Neutrophil (%)	62.247 ± 16.221 (N = 194)	61.245 ± 15.603 (N = 49)	0.576
Lymphocyte (%)	27.012 ± 14.343 (N = 194)	29.333 ± 14.167 (N = 49)	0.309
Monocyte (%)	6.362 ± 3.260 (N = 194)	5.708 ± 2.639 (N = 49)	0.190
Eosinophil (%)	2.673 ± 2.814 (N = 192)	2.596 ± 2.489 (N = 49)	0.756
AST (U/L)	69.840 ± 90.570 (N = 184)	75.213 ± 100.668 (N = 47)	0.864
ALT (U/L)	81.910 ± 101.234 (N = 166)	83.800 ± 113.545 (N = 44)	0.907
CRP (mg/L)	94.998 ± 79.218 (N = 193)	98.827 ± 78.263 (N = 49)	0.605
Albumin (g/dL)	3.256 ± 0.648 (N = 151)	3.571 ± 0.546 (N = 38)	0.006*
Pyuria	40/121 (33.1%)	10/37 (27.0%)	0.490

All continuous lab values are presented as mean ± standard deviation. An asterisk denotes *p*-values of < 0.05. AST: Aspartate transaminase, ALT: Alanine transaminase, CRP: C-reactive protein.

**Fig 1:**
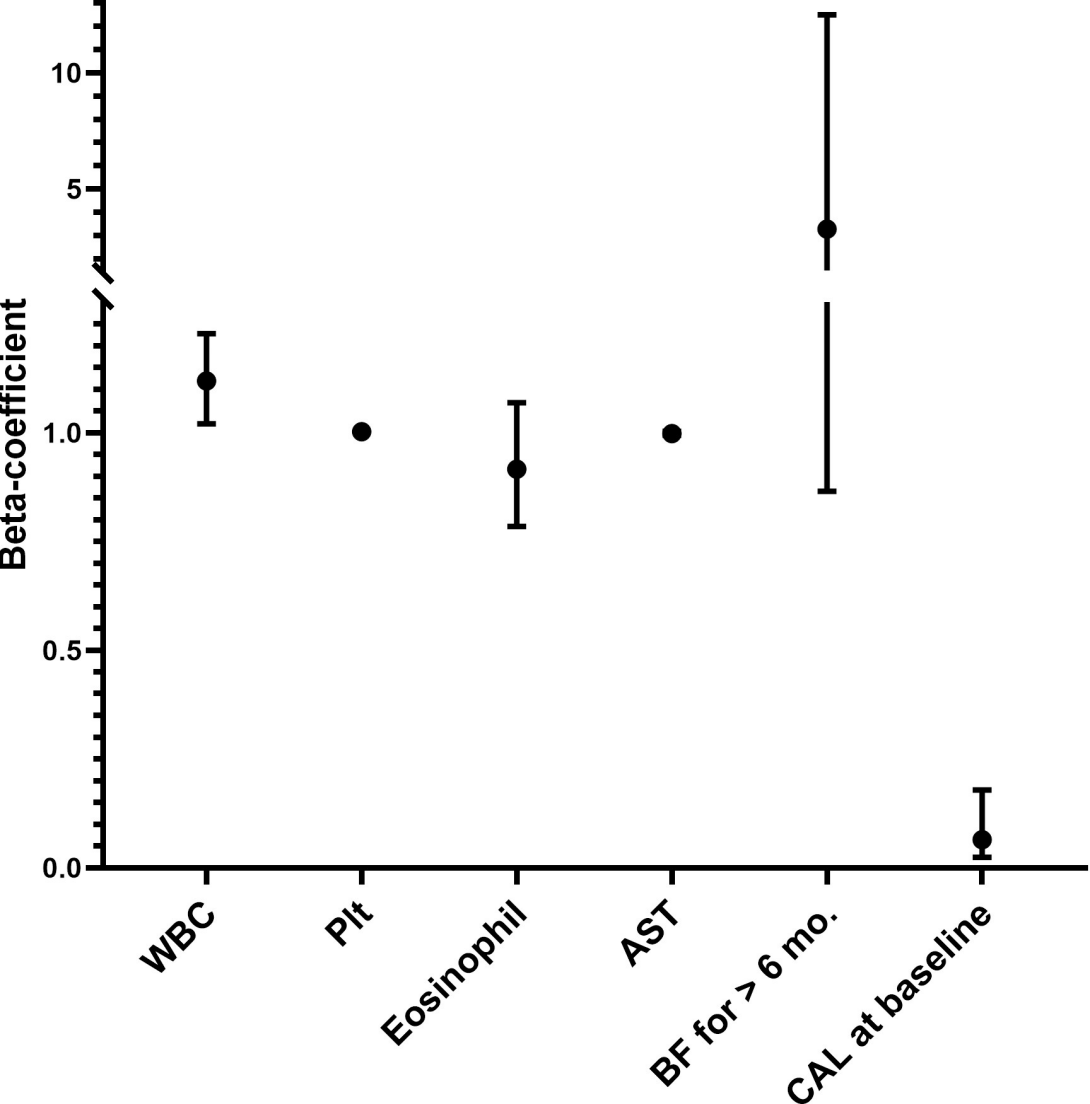
Graphic representation of β-coefficients of all factors included in the regression model. Plotted 95% confidence intervals of the following factors: White cell count (1000/cmm3), platelet count (1000/cmm3), eosinophil percentage (%), aspartate aminotransferase (U/L), breastfeeding for ≥ 6 months and coronary artery lesion at baseline.

We then performed univariate analysis and found the that six factors were significantly associated with the development of coronary artery lesions within our data set. Patients who developed coronary artery lesions were less likely to have been breastfed for at least six months (9.1% Vs. 22.9%, *p* = 0.039), more likely to have coronary artery lesions at baseline (45.5% Vs. 5.4%, *p* < 0.001), a higher white blood cell count (16.167 ± 5.344 Vs. 13.364 ± 4.441 1000/cmm^3^, p = 0.003), a higher platelet count (372.260 ± 148.712 Vs. 317.722 ± 121.124 1000/cmm^3^, *p* = 0.015), a lower eosinophil percentage (1.943 ± 2.716 Vs. 2.800 ± 2.737%, *p* = 0.012), and lower aspartate transaminase values (59.135 ± 86.809 Vs. 73.183 ± 93.603 U/L, *p* = 0.038) (full data can be seen in our S1 File). We then performed and regression of these six factors which were identified on univariate analysis, and found that only the presence of coronary artery lesions at baseline (*β-*coefficient = 0.065, p < 0.001) and white blood count (*β*-coefficient = 0.065, p = 0.018) remained significant after multivariate regression analysis. Breastfeeding for at least 6 months did not remain statistically significant after regression analysis (*β*-coefficient = 3.286, p = 0.081) (Table 3, Fig 1).

**Table 3:** Multivariate regression analysis of demographic factors predicting CAL formation in KD patients.

	β-Coefficient	95% Confidence Interval for β-Coeffeicient	Standard Error	*p-*value
White cell count (1000/cmm3)	1.119	1.020–1.227	0.047	0.018*
Platelet count (1000/cmm3)	1.002	0.999–1.005	0.002	0.223
Eosinophil (%)	0.916	0.784 – 1.069	0.079	0.264
AST (U/L)	0.998	0.994 – 1.003	0.002	0.484
Breastfeeding for ≥ 6 months	3.286	0.865 – 12.491	0.681	0.081
Coronary artery lesion at baseline	0.065	0.024 – 0.179	0.514	< 0.001*

An asterisk denotes p-values of < 0.05. AST: Aspartate transaminase.

## Discussion

In this study, we examined the association between breastfeeding for 6 months or more and clinical outcomes including total duration of fever, IVIG resistance, and CAL formation in patients with KD. We observed that breastfeeding for 6 months or more was associated with a shorter total duration of fever and a lower risk of persistent CAL formation lasting one month or more after disease onset on univariate analysis. To better understand the possible mechanism behind the beneficial effects of breastfeeding may have in patients with KD, we also compared the clinical and laboratory data of KD patients who were breastfed for 6 months or more with those who were not. We found that breastfeeding for 6 months or more was associated with shorter total duration of fever and higher albumin levels prior to IVIG therapy.\. The shorter total duration of fever and higher levels of albumin in KD patients who were breastfed for 6 months or more may point to the potential anti-inflammatory effect of breastfeeding after birth.

In addition, we also found that KD patients who were breastfed for 6 months or more were more likely to present with BCG inoculation site erythema. In countries such as Taiwan where BCG inoculation is a part of the national vaccination program, erythema at the BCG inoculation site is a common and specific feature of patients with KD [21]. While studies regarding the effect breastfeeding on BCG immunity are rare, one study has found that breastfeeding significantly increases lymphocyte proliferation when stimulated by purified protein derivative of Mycobacterium tuberculosis in patients who were inoculated with BCG at birth [22].

To determine whether other demographic factors may also contribute to the development of persistent CALs lasting for one month or more, we performed a multivariate logistic regression of six variables and found that only coronary artery lesions found at baseline and white blood cell count were the most significant predictive factors for persistent CAL formation. Although breastfeeding for 6 months or more was associated with a decreased risk of persistent CAL formation on univariate analysis, it is possible that the protective effect of breastfeeding was modest when compared with other factors. There are several possible reasons why breastfeeding may protect against the development of KD and confer positive outcomes. Breast milk facilitates the establishment of probiotic bacteria in the infantile gut which stimulates T-helper (Th) cells to switch from the Th2-predominant intrauterine immunity to a more mature Th1/Th2 balanced immunity [23]. Previous studies have found that CAL formation in patients with KD is associated with elevated levels of pro-inflammatory Th1 cytokines such as interleukin 6, interferon gamma and tumor necrosis factor alpha [24] and lower levels of Th2 cytokines such as interleukin 5 [25]. KD patients who are breastfed early in life may develop a more mature and appropriate Th1/Th2 response that confers protection against CAL formation. In addition, both short chain fatty acids found directly in breast milk and the probiotic bacterial species such as Lactobaccillus found in the gut of breastfed infants also activates T-regulatory cells (Treg cells) [23]. Breastfeeding may have a more robust anti-inflammatory T-regulatory cell response that protects against the development of KD and CAL formation.

To date, there have been three studies that have specifically examined the effect of breastfeeding on incidence rates of KD, two of which also investigated whether breastfeeding had any effect on important KD outcomes including IVIG resistance and development of coronary artery lesions. The first of these studies was published in 2016 and included data from a population-based longitudinal survey of 37,630 Japanese children. Parents were questioned about infant feeding practices at 6 to 7 months of age, and admission due KD from 6 to 30 months of age was the primary outcome. The authors found that both children who were exclusively and partially breastfed had a lower risk of admission for KD (odds ratio: 0.26 and 0.27, respectively) (14). Subsequently two case-control studies were published which compared the breastfeeding status of patients with KD with age and gender-matched controls. In a study of KD patients identified from a population-based data set of German children from 2012 to 2014, patients with KD were breastfed for a significantly shorter period of time after birth. Breastfeeding duration did not have any effect of the development of coronary artery lesions, or IVIG resistance [26]. In another case-control single center study of 389 Chinese children with KD and 426 gender and age-matched controls found that exclusive breastfeeding appeared to decrease the risk of developing KD (Odds ratio 0.53) but again was not associated with differences in IVIG resistance or risk of developing coronary artery lesions [27].

Many limitations of our study stem from the limited data obtainable on retrospective chart review. For one, patients were considered to have been breastfed for at least 6 months according documentation on chart review, most commonly if the child was still breastfeeding at the 6-month well-child visit. Due to a nationwide policy push to increase breastfeeding rates in Taiwan beginning in 1992, breastfeeding initiation rates during hospitalization after birth is currently quite high, around 85.6% in a nationwide survey performed in 2011 [28]. Therefore, it seems reasonable to assume that patients who were still breastfed by the 6-month visit were indeed breastfed since birth. However, we could not determine if the patient were exclusively or partially breastfed, nor could we accurately determine the exact duration of breastfeeding if the patient was breastfed for less than 6 months. Although the majority of the patients included in this study were not born in our hospital, it is highly likely that many of the 198 (79.5%) patients who received less than 6 months of breastfeeding on our chart review actually received breastfeeding during hospitalization, but did not continue breastfeeding by the time they returned to clinic for their 6-month checkup. Moreover, documentation of partial or exclusive breastfeeding was often unclear, although in a large longitudinal, population-based study in Japan, both partial and exclusive breastfeeding was found to have a protective affect against the development of KD [16].

Another limitation is the small sample size of our study. As a single-center retrospective study, we could only include KD patients who were previously admitted to our hospital and that had documentation of breastfeeding on chart review. Our small sample size might have also been affected by the low rates of breastfeeding in Taiwan [29]. Moreover, patients did not receive echocardiography from the same cardiologist each time, which is customary at our hospital. Due to the relatively high patient numbers and limited number of pediatric cardiologists at our hospital, all children in this study received echocardiography from the cardiologist on duty at that time. However, coronary artery luminal diameters were measured at the specified segments as mentioned before in the methods portion of this manuscript in an effort to decrease inter-operator discrepancies. Other demographic factors that may be associated with breastfeeding including maternal education levels, socio-economic status, maternal smoking, and interval between the onset of KD and last vaccination could not be fully elicited from chart review, and were not included in our study.

Another limitation may be the time period within which the data was collected from 1999 to 2013, which may not reflect changes in current diagnosis and management of KD. Nonetheless, all patients included in our study fulfilled current diagnostic criteria for typical KD, and received at least one dose of IVIG 2g/kg as outlined in the most recent AHA guidelines published in 2017 [18]. Newer therapies have emerged in the years since this study was conducted, including most notably, infliximab, an anti-TNF-α monoclonal antibody, which is currently under phase 3 trials for refractory KD [30]. None of the patients with refractory KD in our study received biological agents, which may limit the applicability of our findings in the era of target therapy.

Further investigation of the effect of breastfeeding on KD disease outcomes would benefit from larger prospective or population based studies, such as the study performed by Yorifugi et al., [16] who were able to longitudinally collect percentage of hospital admissions due to KD, complete demographic data and breastfeeding practices of 37,630 children in a nationwide survey in Japan.

## Conclusions

This is the first study that examines the effect of breastfeeding on the clinical outcomes in patients with KD. We found that breastfeeding for 6 months or more after birth was associated with shorter total duration of fever and a lower percentage of persistent CAL formation that lasted for one month or more on univariate analysis. Further stepwise multivariate logistic regression of demographic variables revealed that coronary artery lesions found at baseline and white blood cell count were the most significant predictive factors predicting persistent CAL formation. Our results suggest that breastfeeding may confer beneficial outcomes in patients with KD, although its effect may be modest in comparison with other factors. Further prospective or population-based studies are needed to further elucidate the effect of breastfeeding on disease outcomes in the patients withKD.

## Supporting information

S1 File Contains Supporting tables Tables 1–3. (DOCX)

S1 Dataset Contains original dataset used in this study. (XLSX)

## Abbreviations

KD: Kawasaki disease
IVIG: intravenous immunoglobulin
CALs: coronary artery lesions
BCG: Bacillus Calmette-Guérin
AST: aspartate transaminase
ALT: alanine transaminase
CRP: C-reactive protein
Th cells: T-helper cells
Treg cells: T-regulatory cells
