# Supplementary material for: Effect of breastfeeding for 6 months on disease outcomes in patients with Kawasaki disease
Source: PLoS One. 2021 Dec 21;16(12):e0261156. doi: 10.1371/journal.pone.0261156 (PMC8691627; doi:10.1371/journal.pone.0261156)
Supplement: S1 File — (DOCX) [file pone.0261156.s001.docx]

Table 1: Comparison of demographic and clinical data between KD patients with coronary artery lesions and those without

|  | No coronary artery lesions (N=205) | Presence of coronary artery lesions (N=44) | *p*-value |
| --- | --- | --- | --- |
| Male Sex | 122/205 (59.5 %) | 31/44 (70.5 %) | 0.176 |
| Age at time of KD diagnosis (years) | 1.932 ± 1.640 (N= 205) | 2.165 ± 1.995 (N=44) | 0.543 |
| Maternal age at patient birth (years) | 30.180 ± 4.676 (N=197) | 30.630 ± 3.904  (N= 43) | 0.589 |
| Day of fever IVIG was given (days) | 5.750 ± 1.737 (N=201) | 6.880 ± 3.201  (N= 42) | 0.077 |
| Total duration of fever (days) | 6.500 ± 2.033 (N=204) | 7.800 ± 3.621  (N= 41) | 0.120 |
| Breastfeeding for ≥ 6 months | 47/205 (22.9 %) | 4/44 (9.1 %) | 0.039* |
| Non-suppurative conjunctivitis | 130/205 (63.4 %) | 33/44 (75 %) | 0.143 |
| Oral mucosa changes | 181/205 (88.3 %) | 38/44 (86.4 %) | 0.721 |
| Lymphadenopathy | 39/205 (19.0 %) | 10/44 (22.7 %) | 0.575 |
| Polymorphous rash | 171/205 (83.4 %) | 36/44 (81.8 %) | 0.797 |
| Changes in the hands and feet | 137/205 (66.8 %) | 30/44 (68.2 %) | 0.862 |
| Erythema around BCG site | 53/175 (30.3 %) | 8/34 (23.5 %) | 0.428 |
| IVIG resistance | 18/205 (8.8 %) | 5/44 (11.4 %) | 0.571 |
| Pericardial effusion | 5/205 (2.4 %) | 3/44 (6.8 %) | 0.151 |
| Coronary artery lesion at baseline | 11/205 (5.4 %) | 20/44 (45.5 %) | <0.001* |

All continuous lab values are presented as mean ± standard deviation. An asterisk denotes *p*-values of < 0.05. BGC: Bacillus Calmette-Guérin, IVIG: Intravenous immunoglobulin.

Table 2: Comparison of laboratory data between KD patients with coronary artery lesions and those without

|  | No coronary artery lesions (N=205) | Presence of coronary artery lesions (N=44) | *p*-value |
| --- | --- | --- | --- |
| White cell count (1000/cmm^3^) | 13.364 ± 4.441  (N = 203) | 16.167 ± 5.344  (N = 40) | 0.003* |
| Hemoglobulin (g/dL) | 11.029 ± 1.117  (N = 203) | 10.932 ± 1.461  (N = 40) | 0.637 |
| Platelet count (1000/cmm^3^) | 317.722 ± 121.124  (N = 203) | 372.260 ± 148.712  (N = 40) | 0.015* |
| Segmented Neutrophil (%) | 62.303 ± 15.611  (N = 203) | 60.732 ± 2.908  (N = 40) | 0.689 |
| Lymphocyte (%) | 27.404 ± 14.118  (N = 203) | 27.865 ± 2.438  (N = 40) | 0.855 |
| Monocyte (%) | 6.148 ± 3.128  (N = 203) | 6.645 ± 3.278  (N = 40) | 0.316 |
| Eosinophil (%) | 2.800 ± 2.737  (N =203) | 1.943 ± 2.716  (N = 40) | 0.012* |
| AST (U/L) | 73.183 ± 93.603  (N = 194) | 59.135 ± 86.809  (N = 37) | 0.038* |
| ALT (U/L) | 82.858 ± 102.936  (N=176) | 79.441 ± 108.836  (N=34) | 0.503 |
| CRP (mg/L) | 95.984 ± 77.979  (N = 202) | 94.708 ± 84.309  (N = 40) | 0.672 |
| Albumin (g/dL) | 3.317 ± 0.634  (N=158) | 3.329 ± 0.686  (N=31) | 0.883 |
| Pyuria | 43/135 (31.9%) | 7/23 (30.4%) | 0.893 |

All continuous lab values are presented as mean ± standard deviation. An asterisk denotes *p*-values of < 0.05. AST: aspartate transaminase, ALT: alanine transaminase, CRP: C-reactive protein.

Table 3: Multivariate regression analysis of demographic factors predicting CAL formation in KD patients

|  | β-Coefficient | 95% Confidence Interval for β-Coeffeicient | | Standard Error | | *p-*value |
| --- | --- | --- | --- | --- | --- | --- |
| White cell count (1000/cmm3) | 1.119 | 1.020 - 1.227 | 0.047 | | 0.018* | |
| Platelet count (1000/cmm3) | 1.002 | 0.999 - 1.005 | 0.002 | | 0.223 | |
| Eosinophil (%) | 0.916 | 0.784 – 1.069 | 0.079 | | 0.264 | |
| AST (U/L) | 0.998 | 0.994 – 1.003 | 0.002 | | 0.484 | |
| Breastfeeding for ≥ 6 months | 3.286 | 0.865 – 12.491 | 0.681 | | 0.081 | |
| Coronary artery lesion at baseline | 0.065 | 0.024 – 0.179 | 0.514 | | < 0.001* | |

An asterisk denotes p-values of < 0.05. AST: aspartate transaminase.
